# Supplementary material for: Magnitude of syphilis sero-status and associated factors among pregnant women attending antenatal care in Jinka town public health facilities, Southern Ethiopia, 2020
Source: PLoS One. 2021 Sep 10;16(9):e0257290. doi: 10.1371/journal.pone.0257290 (PMC8432762; doi:10.1371/journal.pone.0257290)
Supplement: S1 File — (DOCX) [file pone.0257290.s001.docx]

**Questionnaire**

CODE______________________ Date ______________ Name of Facility______________

|  | **Part I: SOCIO - DEMOGRAPHIC DATA** |  |
| --- | --- | --- |
| S NO | Questions | Response |
| 1. | Age | _________years |
| 2. | What is your marital status? | 1. Married  2. Living together  3. Divorced/Separated  4. Widowed  5. Single |
| 3. | Religion | 1. Orthodox  2. Muslim  3. Protestant  4. Other( specify)_______ |
| 4. | Residence | 1. Urban  2. Rural |
| 5. | Do your partner have/had a wife other than you? | 1.yes  2. no |
| 6 | What is your educational status? | 1. unable to read and write  2. Read and write  3. Primary  4. Secondary(9-12)  5. college and above |
| 7 | Husband(partner) educational status | 1. unable to read and write  2. Read and write  3. Primary  4. Secondary(9-12)  5. college and above |
| 8 | What is your occupational status? | 1. Merchant  2. Government employee  3. Private employee  4. Farmer  5. Student  6. Daily laborer  7. Other (specify) |
| 9 | What is occupation of your husband? | 1. Merchant  2. Government employee  3. Private employee  4. Farmer  5. Student  6. Daily laborer  7. Other (specify) |
| 10 | Monthly income of the family (ETB)? | _____________ |
| S No | Part II: **Obstetric history** |  |
| 1. | How many pregnancies you experienced including the current pregnancy? | ___________in number |
| 2. | Do you have a history of ANC visit in the previous pregnancy (for multigravida women) | 1.yes  2. no |
| 3. | Gestational period at the time of screening for syphilis | ___________in month/week |
| 4. | Number of ANC visits for the current Pregnancy? | ___________number |
| 5. | Number of children born alive? | ___________ |
| 6. | Do you have an experience of abortion? | 1.yes  2. no |
| 7. | If question number 6 is yes, how many times? | ________ |
| 8. | If question number 6 is yes, type of abortion occurred? | 1. spontaneous  2. induced |
| S No. | **Part III: Medical history** |  |
| 1. | Have you ever been diagnosed with | 1. diabetic Mellitus  2. HIV/AIDS  3. No |
| 2 | Have you ever been diagnosed with an STI? | 1. Yes  2. No |
| 3 | If ques number 2 is yes, When? | ___________ |
| 4. | If ques no. 2 yes, How were you treated? | 1. No treatment  2. Treated with injection  3. Treated with oral medication  4. Herbal medicine |
| S NO | **Part IV: Laboratory results (document review)** |  |
| 1. | Rapid syphilis test result | 1. Reactive  2. Non-reactive |
| 2. | HIV test result | 1. Reactive  2. Non-reactive |
| S No | **Part V: knowledge related factor** |  |
| 1. | Have you ever heard of STIs? | 1.Yes  2. No |
| 2. | If ques no. is yes source of information | 1. Health institution  2. School  3. Television  4. Radio  5. peers/relative/ neighbor  6. Other (specify) _________ |
| 3. | What are the routes of transmission of STIs | 1. unprotected sexual intercourse  2. infected mother to fetus through trans placental  3. during blood transfusion( donation)  4.sharing injection needles  5. others (specify) |
| 4. | Do the following disease are sexually transmitted? | 1. Gonorrhea  2. Syphilis  3. Genital herpes  4. Trichomonas’s  5. HIV/AIDS  6. Chlamydia 7. Hepatitis B and C |
| 5. | What are the symptoms of STI? | 1. Ulcer on the genital area  2. Vaginal discharge  3. pain during sex or when urinating  4. painless red sores  5. Swollen glands 6. Others (specify) |
| 6. | Can STIs be prevented? | 1. Yes  2. No |
| 7. | Do condoms prevent STIs? | 1.Yes  2. No |
| 8. | Is it possible to prevent mother to child transmission of STI by attending ANC clinic for diagnosis and taking the appropriate medication during pregnancy | 1. Yes  2. No |
| 9. | If a person has HIV/AIDS, does he/she have a greater chance of syphilis infection? | 1. Yes  2. No |
| S No | **Part VI: Behavior related factor** |  |
| 1. | Have you ever used alcoholic drinks?  (Cheka, Tela, Katikala, Beer, Wine supermint etc.) | 1.Yes  2. No |
| 2. | Have you drink alcohol in the last 12 months | 1.Yes  2.No |
| 3. | If ques. No. 2 is yes, how many times per month have you drank | 1.Sometimes (once per month)  2.Two to four times per month  3.five or more than five times per month |
| 4. | Have you ever used drugs of abuse? (like Khat, shisha, cannabis, cocaine, tobacco) | 1.Never  2.Some times  3.Two to four times per month  4.five or more than five times per month |
| 5. | Number of sexual partners ever you have | ______________ |
| 6. | In the past 12 months, how many sexual partners have you had? | ______________ |
| 7. | If ques no.6 answer is two or more, did you use a condom during sexual intercourse? | 1. Yes  2. No |
| 8. | If ques no.7 no, the reason for not using | 1. Partner objection  2. Dislike using a condom  3. Ashamed to ask my partner  4. Faithful to my partner  5. Religious prohibition  6. Other (specify)_________ |
