# Supplementary material for: Magnitude of syphilis sero-status and associated factors among pregnant women attending antenatal care in Jinka town public health facilities, Southern Ethiopia, 2020
Source: PLoS One. 2021 Sep 10;16(9):e0257290. doi: 10.1371/journal.pone.0257290 (PMC8432762; doi:10.1371/journal.pone.0257290)
Supplement: Attachment — Submitted filename: point by point response.docx [file pone.0257290.s003.docx]

**Author’s Point-by-Point Response to the Reviewer's and Editors Reports**

**Title**: Magnitude of Syphilis Sero-status And Associated Factors among Pregnant Women Attending Antenatal Care in Jinka Town Public Health Facilities, Southern Ethiopia, 2020

**Corresponding author**: Asmare Getie/ asmaregetie2017@gmail.com

ID: - PONE-D-21-19327

Journal: PLOS ONE
**Point by point response to Reviewers and Editors**

First of all, the authors would like to thank PLOSE ONE Journal editors and the respective reviewers for reviewing our manuscript and providing the necessary comments to be corrected. As per the comments given, we have made corrections point by point to comment. The authors tried to answer all the issues raised by editorial team and reviewers. Please note that we gave our response in blue font color.

Comment 1: please include additional information regarding the survey or questionnaire used in the study and ensure that you have provided sufficient details that others could replicate the analyses.

Response 1: the questionaries’ were putted as supporting information in the manuscript and it was submitted to the journal as supporting information

Comment 2: Please state whether you validated the questionnaire prior to testing on study participants. Please provide details regarding the validation group within the methods section

Response 2: The questionnaire was already validated hand used by other previous studies

Comment 3. Please amend your current ethics statement to address the following concerns: Please explain why written consent was not obtained, how you recorded/documented participant consent, and if the ethics committees/IRBs approved this consent procedure.

Response 3: Thank you for this suggestion, actually it was editorial error, we have taken written consent from the study participants. It was corrected in the manuscript part.

Comment 4. Please note that in order to use the direct billing option the corresponding author must be affiliated with the chosen institute. Please either amend your manuscript to change the affiliation or corresponding author,

Response 4: thank you very much, it was corrected accordingly. The corrected one was appreciated in the manuscript.

Comment 5: In your Data Availability statement, you have not specified where the minimal data set underlying the results described in your manuscript can be found. PLOS defines a study's minimal data set as the underlying data used to reach the conclusions drawn in the manuscript and any additional data required to replicate the reported study findings in their entirety. All PLOS journals require that the minimal data set be made fully available. For more information about our data policy, please see <http://journals.plos.org/plosone/s/data-availability>.

Response 5: thank you very much for this suggestion, the data was mentioned in the manuscript under supporting information and it was uploaded to the journal as supporting information.

Comment 6. We note you have included a table to which you do not refer in the text of your manuscript. Please ensure that you refer to Table 2 in your text; if accepted, production will need this reference to link the reader to the Table.

Response 6: thank you, it is corrected accordingly

Comment 7. Please include a copy of Table 4 which you refer to in your text on page 11

Response 7: thank you, it was corrected as accordingly

Comment 8: The limitations of the work are not stated and discussed in the Discussion section.

Response 8: thank you very much, as per your recommendation the limitations of this study was incorporated in the manuscript part
comment 9: Abbreviations, such as ANC, CS, COR, AOR, although clear, should be clarified at the first time of use.

Response 9: those abbreviations were clarified accordingly
comment 10: Figures 2 & 3 are unnecessary. The information given in the text is enough.

Response 10: thank you very much as per your recommendation figures 2 and 3 were excluded
